# A Noncellulosomal Mannanase26E Contains a CBM59 in *Clostridium cellulovorans*

**DOI:** 10.1155/2014/438787

**Published:** 2014-03-27

**Authors:** Kosuke Yamamoto, Yutaka Tamaru

**Affiliations:** ^1^Department of Life Sciences, Graduate School of Bioresources, Mie University, 1577 Kurimamachiya, Tsu, Mie 514-8507, Japan; ^2^Graduate School of Bioresources, Department of Bioinfomatics, Mie University Life Science Research Center, Mie University, 1577 Kurimamachiya, Tsu, Mie 514-8507, Japan; ^3^Laboratory of Applied Biotechnology, Mie University Industrial Technology Innovation Institute, Mie University, 1577 Kurimamachiya, Tsu, Mie 514-8507, Japan

## Abstract

A multicomponent enzyme-complex prevents efficient degradation of the plant cell wall for biorefinery. In this study, the method of identifying glycoside hydrolases (GHs) to degrade hemicelluloses was demonstrated. The competence of *C. cellulovorans*, which changes to be suitable for degradation of each carbon source, was used for the method. *C. cellulovorans* was cultivated into locust bean gum (LBG) that is composed of galactomannan. The proteins produced by *C. cellulovorans* were separated into either fractions binding to crystalline cellulose or not. Proteins obtained from each fraction were further separated by SDS-PAGE and were stained with Coomassie Brilliant Blue and were detected for mannanase activity. The proteins having the enzymatic activity for LBG were cut out and were identified by mass spectrometry. As a result, four protein bands were classified into glycosyl hydrolase family 26 (GH26) mannanases. One of the identified mannanases, Man26E, contains a carbohydrate-binding module (CBM) family 59, which binds to xylan, mannan, and Avicel. Although mannose and galactose are the same as a hexose, the expression patterns of the proteins from *C. cellulovorans* were quite different. More interestingly, zymogram for mannanase activity showed that Man26E was detected in only LBG medium.

## 1. Introduction

**Figure 1:**
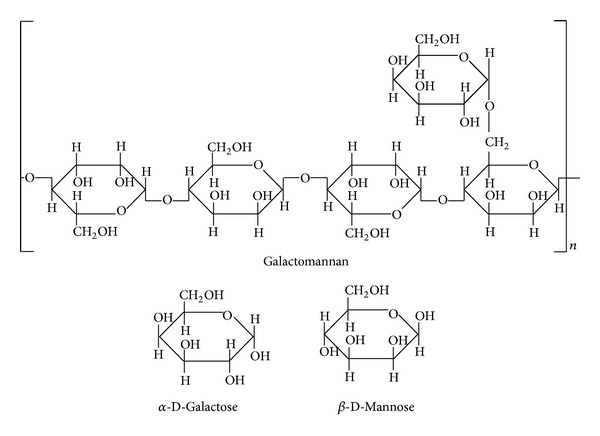
Structural formulas for galactomannan and component sugars. Galactomannan is a polysaccharide consisting of a mannose backbone with galactose side chains: the ratio of mannose : galactose is 4 : 1.

The plant cell wall is composed of cellulose, hemicelluloses, and lignin. The formation of plant cell wall prevents a biomass saccharification for biorefinery [1]. Since hemicellulose was composed of various sugar chains such as glucose, xylose, galactose, mannose, and arabinose, the degradation of plant cell wall needed various and different kinds of enzymes [2]. *β*-mannanase mainly produces mannose to degrade galactomannan or glucomannan. Galactomannan, which is a main component in locust bean gum (LBG), is composed of *β*-(1-4)-D-mannopyranoside chain attached with *α*-D-galactose (Figure 1).

*C. cellulovorans*, which is an anaerobic mesophile [3], has an ability to degrade plant-biomass directly to produce a multienzyme complex called the “cellulosome” [4–8]. Genomic analysis revealed that this organism not only has 17 cellulosomal cellulases and 10 cellulosomal hemicellulases, but it also has 63 noncellulosomal enzymes related to polysaccharides degradation [9, 10]. In addition, members of cellulosomal subunits are changed to suitable and target substrates [11–14]. Thus, the method of functional protein evaluation is the best way to identify the enzymes for degrading each carbohydrate substrates. In this study, we identified several mannanases from* C. cellulovorans* for degrading LBG using functional protein evaluation and genomic data. In addition, we report that one of the identified mannanase 26E (Man26E) contains a carbohydrate-binding module (CBM) family 59.

## 2. Materials and Methods

### 2.1. Bacterial Strains and Media

The proteins used in proteomic and comparative analyses were produced by* C. cellulovorans* 743B. This organismwas grown under strictly anaerobic conditions at 37°C in medium containing 0.5% (w/v) glucose, mannose, galactose, or LBG as a carbon source [3].

### 2.2. Alignment of Amino Acid Sequences and Phylogenetic Trees

The databases used for sequencing data are as follows: NCBI (http://www.ncbi.nlm.nih.gov/), GenomeNet (http://www.genome.jp/), and CAZy (http://www.cazy.org/). The Clustal program [15] was used to carry out alignments of amino acid sequences.

### 2.3. Protein Production and Purification

The culture supernatants in* C. cellulovorans* were centrifuged and the supernatant was collected. The proteins in the supernatant were precipitated by dissolving ammonium sulfate and the precipitate was dissolved in 50 mM acetate buffer (pH 6.0). The solution was dialyzed at 4°C for three hours. This solution was used as a total protein fraction. Crystalline cellulose (Avicel) was added to a 1-mL aliquot and incubated at 4°C for 1 hour. The mixture was centrifuged and the supernatant was used as a nonbound fraction. The pellets binding to Avicel were washed twice by the same buffer at 4°C and were centrifuged. After the supernatant was removed, the pellets binding to Avicel were washed by distilled water at 25°C. The supernatant was used as a bound fraction. The concentration of proteins was determined with bovine serum albumin as a standard using protein assay kit (BIO-RAD).

### 2.4. Zymogram

The zymogram for mannanase activity was performed with 7.5% (w/v) polyacrylamide gel containing 0.07% (w/v) LBG. After SDS-PAGE was performed, the gels were soaked into 25% isopropanol at room temperature for 6 h. The gels were washed for 20 min with 50 mM acetate buffer (pH 6.0) and were incubated at 37°C for 1 hour in the same buffer. Next, the gels were stained with 0.2% (w/v) Congo red solution for 20 min. The stained gels were destained by 1 M NaCl several times until white halos emerge.

### 2.5. Functional Protein Evaluation

**Figure 2:**
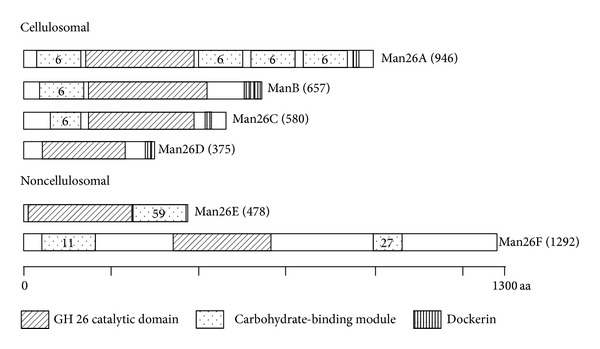
Schematic models for* C. cellulovorans *GH family 26 mannanases. Numbers in the model indicate CBM family number. Protein names are represented on the right side of the model. A length of amino acid sequence is in parentheses.

The purified proteins were separated by SDS-PAGE and were stained by Coomassie brilliant blue (CBB) and for mannanase activity. The selected 5 bands, which were observed by mannanase activity with zymogram, were cut out. The pieces of gels containing protein(s) were alkylated and digested by trypsin (Figure 2). The digested products by typsin were separated to 119 aliquots by nano-LC and the aliquots were analyzed by mass spectrums (MS). The proteins containing the aliquots were identified to compare amino acid sequences. The resulted analysis was performed with genomic data using Mascot software (MATRIX SCIENCE).

## 3. Results and Discussion

### 3.1. Comparison of Man26E and Other GH Family 26 Mannanases in* C. cellulovorans*

**Figure 3:**
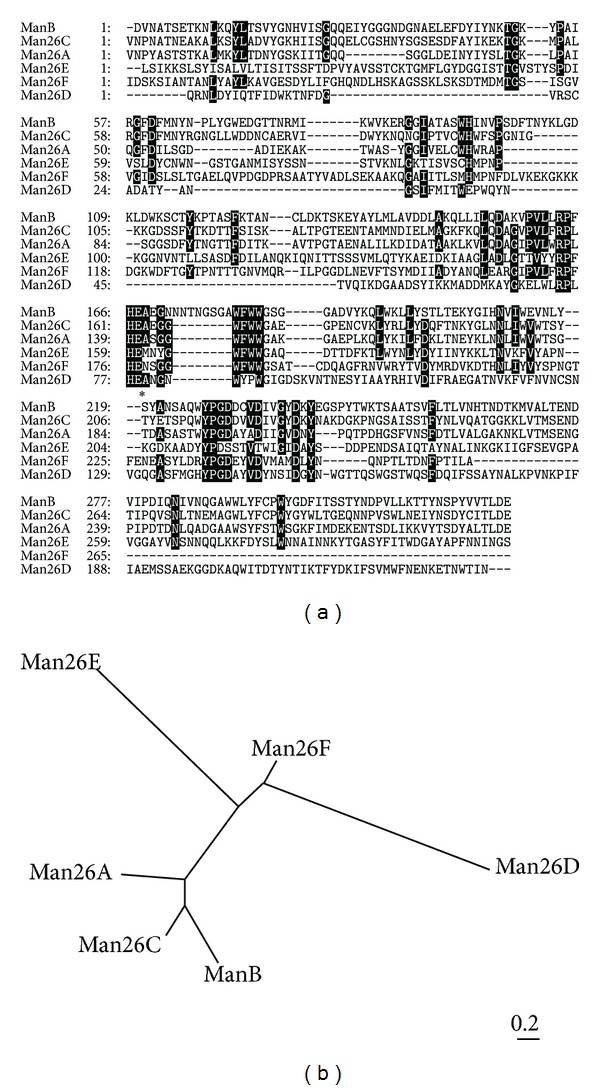
Alignment of catalytic domain of* C. cellulovorans* GH family 26 mannanases (a) and phylogenetic tree based on amino acid sequences of GH family 26 mannanases (b). An amino acid followed by an asterisk indicates the putative catalytic base. Phylogenetic tree was constructed by the neighbour-joining (NJ) method. Bar: 0.2 expected amino acid substitutions per site.

According to* C. cellulovorans* genomic data, schematic models of GH family 26 mannanases are shown in Figure 2. Four GH26 mannanases (Man26A, Man26C, Man26D, and ManB) were identified into cellulosomal enzymes that have a dockerin domain at the C-terminus. In contrast, Man26E and Man26F were classified into noncellulosomal enzymes. The alignment of the catalytic domains among GH26 mannanases is shown in Figure 3(a). Based on amino acid sequences of the catalytic domains among them, phylogenetic trees of GH26 mannanases in* C. cellulovorans* are shown in Figure 3(b). Man26A, Man26C and Man5B [3] have carbohydrate-binding module 6 (CBM6) that were reported for binding amorphous cellulose and *β*-1,4-xylan. On the other hand, Man26F has a CBM11 binding of *β*-1,4-glucan and *β*-1,3-1,4-mixed linked glucans and a part of CBM27 binding to mannan, respectively. Putative catalytic base [16, 17], which is Glu-166 in Man26E, was conserved in all GH26 mannanases in* C. cellulovorans* (Figure 3(a)). Interestingly, phylogenetic tree indicated that Man26A, Man5B, and Man26C were closely located, while Man26D, Man26E, or Man26F was far from each other (Figure 3(b)). These results suggested that three genes encoding: Man26A, Man5B, and Man26C were close to each other because there enzymes are cellulosomal enzymes. Moreover, it is possible that the genes encoding Man26D, Man26E, and Man26F might be obtained from other organisms except* Clostridia*.

### 3.2. Functional Protein Evaluation and Induced Expression of Man26E Caused by the Difference of Carbon Source

**Figure 4:**
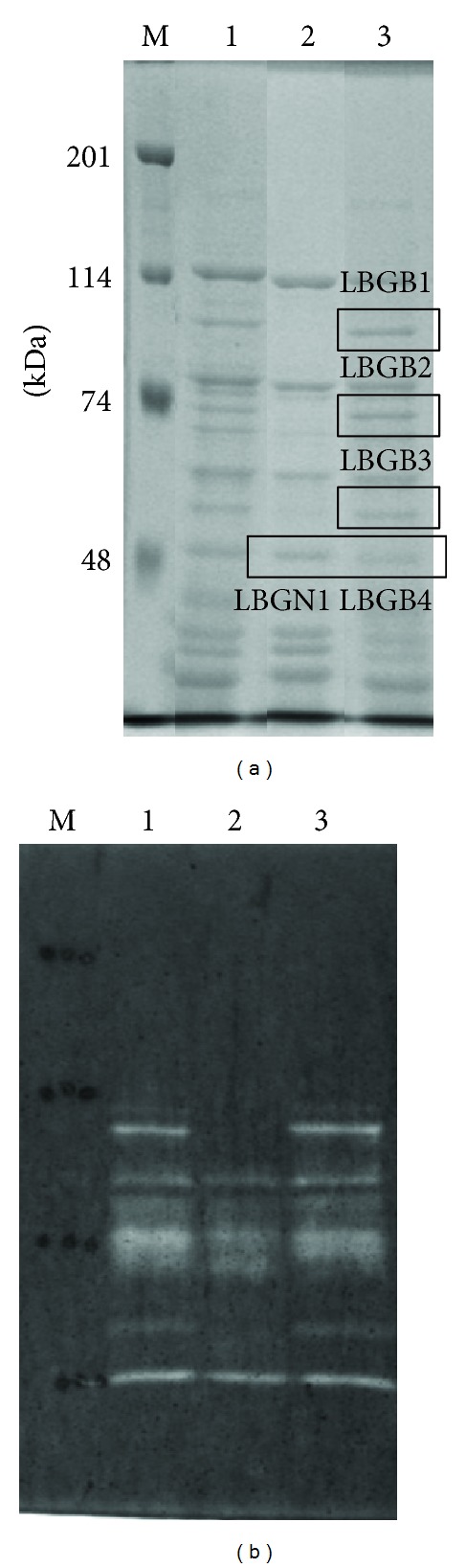
SDS-PAGE analysis (a) zymogram (b) of the proteins from* C. cellulovorans *grown on LBG. The protein bands in a square were cut out for functional protein evaluation. SDS-PAGE was performed with a 7.5% polyacrylamide gel. Zymogram was carried out with a 7.5% polyacrylamide gel containing 0.07% LBG. Lane M, protein molecular mass standards (molecular masses shown in the left); lane 1, total protein fraction; lane 2, Avicel-nonbound fraction; lane 3, Avicel-bound fraction.

Table 1 shows the identified mannanases by functional protein evaluation. The protein bands that are represented to LBGB1 and LBGB3 contained Man26A and ManB [18], respectively (Figure 4), while LBGB4 and LBGN1 contained Man26E. On the other hand, LBGB1 contained Eng5B (accession number: YP_003842513.1.) which belongs to GH5. More interestingly, all these enzymes except Eng5B in the LBG media had a GH26 region.

**Figure 5:**
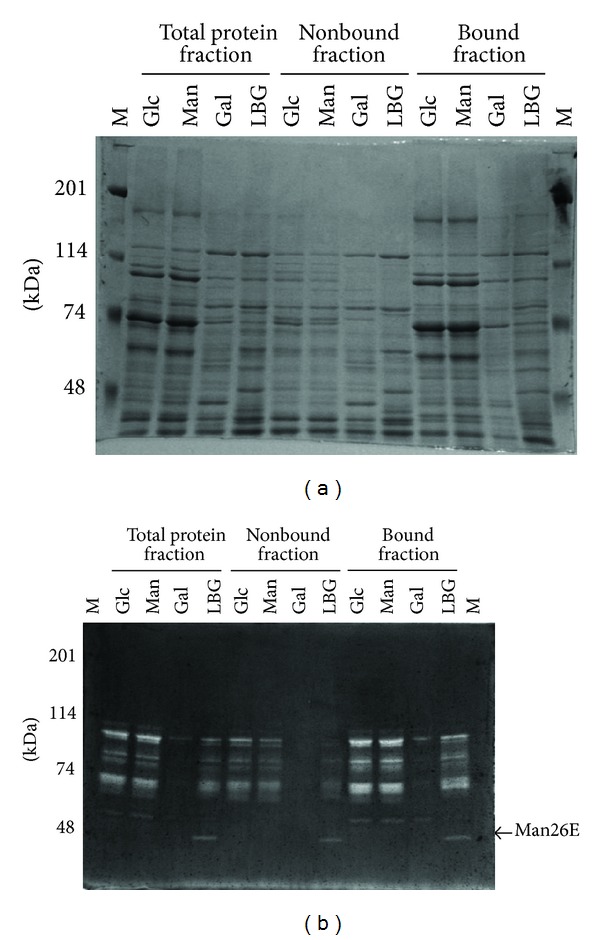
SDS-PAGE analysis of total, nonbound and bound fractions obtained from cells grown on different carbon sources. After electrophoreses, the gels were stained by Coomassie brilliant blue (a) and were detected for mannanase activity (b). M, protein molecular mass standard (molecular masses shown at the left); Glc, D-glucose; Man, D-mannose; Gal, D-galactose; LBG, Locust bean gum.

*C. cellulovorans* can be grown on glucose, mannose, galactose, or LBG. The proteins from the culture supernatants were subjected to SDS-PAGE. There was no difference of bands in all fractions between glucose and mannose as a hexose (Figure 5(a)). On the other hand, several different bands appeared between galactose and LBG in comparison with glucose and mannose. In particular, only few bands having mannanase activity were detected with galactose (Figure 5(b)). More interestingly, although LBG is comprised of mannose and galactose, Man26E band appeared in only LBG in all fractions.

### 3.3. Man26E Contains a CBM59

**Figure 6:**
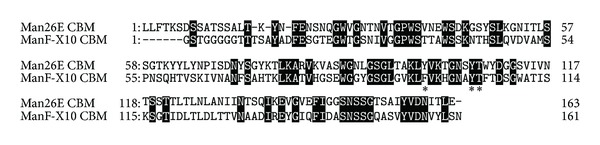
Alignment of CBMs in Man26E from* C. cellulovorans* and ManF-X10 from uncultured bacteria.**:** Aromatic amino acids that are estimated to bind carbohydrate substrates are marked with an asterisk.

**Figure 7:**
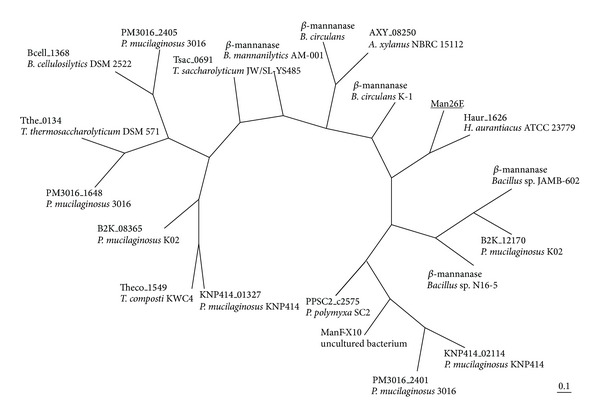
Phylogenetic trees based on amino acid sequences of CBM family 59. The tree was constructed by the neighbour-joining (NJ) method. Abbreviated generic name was described as follows:* Amphibacillus xylanus *NBRC 15112,* Bacillus cellulosilyticus *DSM 2522,* Bacillus circulans*,* Bacillus circulans *K-1,* Bacillus mannanilyticus, Herpetosiphon aurantiacus* ATCC,* Paenibacillus mucilaginosus *3016,* Paenibacillus mucilaginosus *K02,* Paenibacillus mucilaginosus *KNP414,* Paenibacillus polymyxa *SC2,* Thermoanaerobacterium saccharolyticum *JW/SL-YS485,* Thermoanaerobacterium thermosaccharolyticum* DSM 571, and* Thermobacillus composti *KWC4. Bar: 0.1 expected amino acid substitutions per site.

Amino acid sequence analysis indicated Man26E has a CBM59 at its C-terminal region (Figure 6). The homologies between 163 amino acid sequence of CBM59 in Man26E and the other amino acid sequences were, for example, as follows: 53% with mannan endo-1,4-beta-mannosidase A and B from* Paenibacillus mucilaginosus* 3016; 52% with glycoside hydrolase families 5 and 6 from* Paenibacillus polymyxa* SC2; 51% with mannanase from* Bacillus circulans*; 48% with beta-mannanase precursor from* Bacillus *sp. N16-5; 48% with mannanase from* Bacillus* sp. JAMB-602; 41% with xylanase from uncultured bacterium. Phylogenetic tree showed that there were no* Clostridia* possessing a CBM59 except for* C. cellulovorans *(Figure 7). All of 15 organisms (containing uncharacterized organisms) having a CBM59 were bacteria and firmicutes outside of* Herpetosiphon aurantiacus*, which is classified into chloroflexi. Interestingly, CBM59 of* C. cellulovorans *was most close to* H. aurantiacus* CBM59 between the evolutionary relationships (Figure 7). The xylanase ManF-X10 from uncultured bacterium sharing 41% identity with the C-terminal region of Man26E was demonstrated to bind ivory nut mannan, oat spelt xylan and Avicel [19]. However, it is suggested that the Avicel-bound activity of Man26E showed only nonbound fractions (Figures 4 and 5(b)). Furthermore, although Man26A, Man5B, and ManB were detected with an Avicel-bound fraction, Man26E was identified into a nonbound fraction (Figure 4 and Table 1). Putative aromatic residues of binding to the carbohydrate substrates were conserved in three-fourths of aromatic residues between ManF-X10 and Man26E (Figure 6). These results indicated that the affinity of CBM59 in Man26E to polysaccharides such as cellulose and hemicelluloses was weakened by deletion of aromatic amino acids.

Since CBM59 in Man26E binds to LBG and Avicel, Man26E could play a role on degradation of mannan attached to cellulose in the plant cell wall. These results in this study were strongly supported by previous studies that a high-synergistic effect was generated between the cellulosome and noncellulosomal enzymes [20]. For example, it is known that the main scaffolding protein CbpA in the* C. cellulovorans* cellulosome binds to crystalline cellulose. Therefore, it is assumed that Man26E should be close to the cellulosome and could contribute to degrade the plant cell wall such as LBG with synergistic activity.

## 4. Conclusions

Man26E, which was one of the identified mannanases from the culture supernatant of* C. cellulovorans* by functional protein evaluation, was expressed only in LBG consisting of mannose and galactose, whereas it was not induced into glucose, mannose, or galactose. The alignment of CBM59 in Man26E revealed that aromatic amino acids were highly conserved and estimated to bind carbohydrate substrates. This is the first report that Man26E in* C. cellulovorans* contains a CBM59 which has never been found in* Clostridia*.

## Figures and Tables

**Table 1:** Identified mannanases by functional protein evaluation.

Band name	Accession number	GH family number	CBM family number	Dockerin	Name
LBGB1	YP_003845544.1	26	6	+	Man26A
LBGB1	YP_003844553.1	5	11	+	Man5B
LBGB3	YP_003844078.1	26	6	+	ManB
LBGB4	YP_003845549.1	26	59	−	Man26E
LBGN1	YP_003845549.1	26	59	−	Man26E
